# Revitalizing old age: impact of a cognitive stimulation programme on emotional wellbeing and cognitive-executive functioning in older adults from Tacna, Peru

**DOI:** 10.3389/fpubh.2026.1876477

**Published:** 2026-07-29

**Authors:** María Soledad Porras-Roque, Kevin Mario Laura-De La Cruz, Olga Giovanna Valderrama Ríos

**Affiliations:** 1 Facultad de Ciencias de la Salud, Universidad Nacional Jorge Basadre Grohmann, Tacna, Peru; 2 Facultad de Educación, Comunicación y Humanidades, Universidad Nacional Jorge Basadre Grohmann, Tacna, Peru; 3 Facultad de Ciencias de la Salud, Universidad Nacional del Callao, Callao, Peru

**Keywords:** active aging, cognitive functioning, cognitive stimulation, emotional wellbeing, executive functions, older adults, Peru

## Abstract

**Background:**

Population aging and the growing burden of cognitive decline make community-based non-pharmacological interventions increasingly relevant for older adults. However, evidence from Peruvian community settings remains limited, and many local programmes combine screening with activities whose cognitive and emotional components are insufficiently described.

**Objective:**

To evaluate the pre-post impact of a structured cognitive stimulation programme on cognitive status, executive functioning, programme participation and immediate emotional perception among older adults in Tacna, Peru, in 2025.

**Methods:**

An applied, quantitative, quasi-experimental one-group pre-test-post-test study was conducted in community settings. The accessible population comprised 450 older adults, from which 217 participants aged 60 years or older were selected through stratified random sampling. The intervention lasted 6 months and included 60-min sessions two to three times per week in small groups. Eight modules stimulated attention, memory, language, calculation, executive functions, gnosias, praxias and graphomotor skills. Cognitive status was assessed with the Short Portable Mental Status Questionnaire/Pfeiffer Scale and the Montreal Cognitive Assessment (MoCA), while executive functioning was assessed with the INECO Frontal Screening (IFS). Monitoring and satisfaction forms recorded participation, module acceptance and immediate emotional perception.

**Results:**

Mean MoCA scores increased from 18.06 to 26.66, mean IFS scores increased from 14.62 to 24.50 and mean Pfeiffer errors decreased from 2.11 to 0.54. Wilcoxon signed-rank tests showed statistically significant pre-post differences for all three measures (*p* < 0.001), with the same direction of change across central, northern and southern implementation zones. Emotional-perception records showed an increase in the “happy” response from 96.60% before module activities to 99.87% after activities, while sadness decreased from 3.33 to 0.13%.

**Conclusion:**

The programme was associated with statistically significant short-term improvements in cognitive screening scores and executive functioning, together with high acceptability and a favorable pattern of immediate emotional perception. Because the design lacked a control group and used screening rather than comprehensive neuropsychological assessment, the findings should be interpreted as evidence of pre-post improvement and feasibility, not as definitive causal proof.

## 1 Introduction

Population aging is a central public-health transformation because the World Health Organization estimates that by 2030 one in six people worldwide will be aged 60 years or older, and that the population aged 60 years or older will double to approximately 2.1 billion by 2050 (1). This demographic transition is particularly significant for Latin America and Peru, as aging societies necessitate a shift toward care models that emphasize the preservation of autonomy, social participation, and intrinsic capacity, rather than focusing exclusively on disease treatment (2).

Cognitive health is functionally important in later life because it refers to the ability to think, learn and remember clearly, capacities that are necessary for everyday tasks such as medication management, financial decisions, cooking and independent mobility (3). Dementia illustrates the magnitude of the challenge because 57 million people were living with dementia worldwide in 2021, and dementia is among the leading causes of disability and dependency in older adults (4). Cognitive decline is not an inevitable consequence of aging, since risk is influenced by modifiable factors such as physical inactivity, social isolation, depression, low educational attainment and cognitive inactivity (4). The absence of curative treatment for dementia strengthens the rationale for prevention and risk-reduction strategies, and WHO guidelines explicitly recommend evidence-based lifestyle and intervention approaches to delay or prevent cognitive decline and dementia (5).

Cognitive reserve provides a useful explanatory framework because it describes the adaptive capacity of cognitive processes that allows some people to maintain functioning despite age-related or disease-related brain changes (6). Recent longitudinal synthesis supports this framework, as a systematic review and meta-analysis found that cognitive reserve accumulated in early life, midlife and late life was associated with lower dementia risk, with protective estimates reported for each life-course stage (7). Lifestyle and social participation are also relevant because a population-based study of 2,315 cognitively healthy older adults found that cognitive and social activity, physical activity and healthy diet were positively associated with cognitive function, with cognitive reserve mediating part of this association (8).

Cognitive-based interventions are therefore scientifically justified because a systematic review and meta-analysis of randomized controlled trials reported that these interventions improved cognitive function in older adults and distinguished cognitive stimulation, cognitive training and cognitive rehabilitation as related but different approaches (9). For the present article, the term “cognitive-executive functioning” is used instead of the broader expression “mental functioning” because the study measured cognitive status with the Pfeiffer Scale and MoCA and executive functioning with the INECO Frontal Screening. Emotional wellbeing is considered cautiously in this study because dementia and cognitive impairment may be accompanied or preceded by changes in mood, emotional control, behavior or motivation; however, the present data capture immediate emotional perception during module activities rather than clinical affective symptoms (4).

In Peru, national demographic estimates show a growing older-adult population, and in Tacna this trend increases the need for feasible community interventions that combine early screening, cognitive activation, social participation and follow-up within local care spaces (10). The local problem addressed by this study is that screening can identify possible cognitive risk, but screening alone does not provide stimulation, social engagement or structured practice for preserved abilities. Therefore, the present programme combined baseline cognitive-executive screening, a 6-month structured cognitive stimulation protocol and post-intervention assessment. The objective was to evaluate the impact of the programme on emotional wellbeing and cognitive-executive functioning among older adults in Tacna, Peru, in 2025. The study hypothesis was that participants would show statistically significant improvement between pre-test and post-test cognitive-executive scores after the intervention.

## 2 Methods

### 2.1 Study design

An applied, quantitative, quasi-experimental study with a one-group pre-test-post-test design was conducted. This design was appropriate for evaluating within-participant change after a community intervention, but it does not allow causal inference equivalent to a randomized controlled trial. The study was carried out in community and health-related settings serving older adults in the province of Tacna, Peru, during 2025.

### 2.2 Study setting, population and sample

The universe comprised adults aged 60 years or older residing in Tacna and participating in community spaces or care centers for older adults. The broader reference population at the district level comprised 23,568 older adults from Coronel Gregorio Albarracín Lanchipa, Alto de la Alianza, Ciudad Nueva, Pocollay, and Tacna. The study's accessible population consisted of 450 older adults registered or participating in the selected community settings. From this cohort, 217 participants were selected through stratified random sampling to maintain representativeness across fieldwork zones and subgroups.

Participating centers were grouped into three operational fieldwork zones for descriptive and territorial analysis: central, northern and southern Tacna. This classification was based on the geographical distribution of the community settings and the fieldwork route used by the research team. The zonal analysis was included to test whether the intervention had consistent effects across the different community contexts. Geographic sector differences in access to health services, educational opportunities, social participation and community resources may have contributed to the analysis of cognitive and executive-function outcomes by zone to provide further information on the feasibility, adaptiveness and reliability of the programme under different conditions of implementation. The zone comparisons were secondary and exploratory and were not designed to establish causal territorial differences.

Inclusion criteria were age 60 years or older, either sex, residence in the intervention zone, ability to provide informed consent, cognitive status compatible with understanding and participating in the activities, ranging from intact cognition to mild impairment, and willingness to attend scheduled sessions. Exclusion criteria were advanced neurodegenerative disease, psychiatric disorder or psychological instability likely to interfere with participation, severe communication impairment limiting comprehension or task execution, and unstable or acute medical conditions that could increase participation risk.

### 2.3 Ethical considerations

The study involving human participants was reviewed and approved by the Institutional Ethics Committee of Universidad Nacional Jorge Basadre Grohmann under approval code 2024-059-CEIUNJBG. The approval renewal was issued in Tacna on 14 October 2025, extending the validity of the study protocol until 25 July 2026. Before enrolment, candidates received information about the study objectives, procedures, voluntary participation, confidentiality and the right to withdraw without penalty. All participants provided informed consent prior to participation.

### 2.4 Intervention protocol

The intervention consisted of a structured cognitive stimulation programme implemented over 6 months. Participants attended two to three 60-min sessions per week. Sessions were delivered in small groups of two to four older adults to facilitate interaction, individual monitoring and adaptation to participant needs.

Each session followed an opening, technical and closing sequence. The opening phase included greeting, rapport-building, emotional climate assessment and brief integration dynamics. The technical phase developed the planned module activities through cognitive exercises, brain-gym activities, narration of experiences, eye-hand coordination tasks, verbal association, problem-solving and recreational tasks. The closing phase summarized achievements, reinforced participation and invited participants to the next session. The cognitive stimulation intervention protocol is summarized in Table 1.

**Table 1:** Summary of the cognitive stimulation intervention protocol.

Stage	Module or stage	Objective	Main activities
Baseline	Pre-assessment and integration dynamics	Establish baseline and promote rapport	Application of Pfeiffer/SPMSQ, MoCA and IFS; integration activities.
Module 1	Attention	Develop selective, sustained and divided attention	Warm-up, breathing exercises, rhythm activities, “Simon says,” cancellation tasks, puzzles and visual search exercises.
Module 2	Memory	Stimulate short-term memory, working memory and autobiographical recall	Matching pairs, recall of images and words, association strategies, personal-history narration and delayed recall activities.
Module 3	Language	Strengthen oral expression, comprehension and verbal fluency	Verbal association, vocabulary expansion, reading comprehension, naming activities and narration of experiences.
Module 4	Calculation and numerical reasoning	Reinforce arithmetic, sequencing and practical numerical reasoning	Basic arithmetic, numerical series, money-handling exercises and simple problem solving.
Module 5	Executive functions and logical reasoning	Stimulate planning, inhibition, sequencing and problem solving	Color sequencing, categorization, rule-shifting, planning tasks, inhibition games and everyday-problem analysis.
Module 6	Gnosias	Stimulate recognition through visual, auditory and tactile channels	Recognition of objects, sounds, images, textures and familiar environmental stimuli.
Module 7	Praxias	Strengthen ideomotor, constructive and coordination abilities	Fine and gross motor coordination, imitation of gestures, motor sequences and object-use simulation.
Module 8	Graphomotor skills and post-assessment	Develop graphomotor control and evaluate change	Writing, calligraphy, tracing, eye-hand coordination and final administration of standardized screening tools.

The protocol was adapted from the source thesis intervention schedule and reorganized to show the eight cognitive domains stimulated during implementation.

### 2.5 Measurement instruments

The study used complementary screening instruments because no single brief test captures all relevant dimensions of cognitive-executive functioning. The Pfeiffer Scale/SPMSQ provides a brief estimate of cognitive status through errors in orientation, memory and basic mental operations; lower post-intervention error counts indicate better performance. The MoCA provides global cognitive screening across domains such as attention, memory, language, abstraction, visuospatial/executive skills and orientation; higher scores indicate better performance. The INECO Frontal Screening focuses on executive functions and frontal systems, including inhibitory control, working memory, abstraction and motor programming; higher scores indicate better executive performance.

Trained research personnel administered the Pfeiffer Scale, MoCA, and IFS at both baseline and post-intervention using structured assessment protocols. These instruments were utilized strictly for screening and early detection, rather than to establish a formal clinical diagnosis or to conduct secondary-level neuropsychological assessments. This distinction is critical, as the reported results characterize changes in screening performance rather than comprehensive neuropsychological recovery.

Two supplementary instruments were employed for programme monitoring. The monitoring form tracked participant attendance and engagement throughout the modules, recording baseline involvement in cognitive activities and post-intervention workshop evaluations. The satisfaction form recorded module acceptance and immediate emotional perception before and after activities using simple categories such as happy, sad and angry.

Emotional component was assessed using a short satisfaction and emotional perception form before and after each module activity. Participants were asked to select the emotional state that best described how they were feeling at the moment with one of three categorical responses: happy, sad or angry. The instrument was not a Likert-type scale and was not designed to measure clinical symptoms of depression, anxiety, or psychological distress. Rather it was meant to be a simple monitoring tool to assess participants' immediate emotional responses and acceptability of the programme. Responses were analyzed using frequencies and percentages and compared descriptively between pre-activity and post-activity measurements.

Screening results were classified using functional cut-off values. The Pfeiffer Scale/SPMSQ was scored as follows: 0–2 errors, normal cognition; 3–4 errors, mild impairment; 5–7 errors, moderate impairment; 8–10 errors, severe impairment. For the MoCA, scores < 26 were interpreted as indicating cognitive impairment and scores ≥26 were interpreted as indicating normal cognitive screening performance. A score below 25 on the INECO Frontal Screening (IFS) suggested possible executive dysfunction, while a score of 25 or above suggested preserved executive functioning. The cut points were used for screening classification only and not for clinical diagnosis. The measurement instruments and their operational interpretation are summarized in Table 2.

**Table 2:** Measurement instruments and operational interpretation.

Instrument	Construct assessed	Administration moment	Interpretation in this study
Pfeiffer scale/SPMSQ	Brief cognitive status and errors in orientation/memory	Pre-test and post-test	Fewer errors indicate better cognitive performance.
MoCA	Global cognitive screening	Pre-test and post-test	Higher scores indicate better cognitive screening performance; used as a screening test, not a diagnosis.
INECO frontal screening (IFS)	Executive functioning/frontal systems	Pre-test and post-test	Higher scores indicate better executive functioning.
Monitoring form	Participation and engagement in modules	Throughout the programme	Classifies participation and post-intervention valuation of workshops.
Satisfaction form	Module acceptance and immediate emotional perception	Before and after module activities	Records perceived emotional state and acceptance of each module.

Multiple instruments were used to capture complementary domains rather than duplicate measurement.

### 2.6 Reliability and validity

Internal consistency was examined using Cronbach's alpha. Acceptable values were reported for the pre-test (α = 0.625), Pfeiffer Scale (α = 0.728), MoCA (α = 0.709) and IFS (α = 0.766). Content validity was assessed by expert judgement through indicators of clarity, objectivity, relevance and coherence, with a range proportion coefficient of 0.87, interpreted as high agreement among judges. The construct validity was also tested using the Bartlett's test of sphericity and the Kaiser-Meyer-Olkin (KMO) measure of sampling adequacy. The KMO value obtained was 0.842 which indicates the sampling adequacy is adequate for factor analysis. Bartlett's test of sphericity was statistically significant (χ^2^ = 1,456.38, df = 406, *p* < 0.001), indicating that the correlation matrix was suitable for factor extraction and the instruments had construct validity.

### 2.7 Data collection procedure

Recruitment was conducted through announcements in community centers, health clinics and other places frequently attended by older adults. Interested candidates were screened according to inclusion and exclusion criteria. Baseline assessments were then performed using the selected instruments. The intervention followed the 6-month protocol, and post-intervention assessments were administered at the end of the programme using the same cognitive-executive screening instruments. Emotional perception and satisfaction were recorded during module implementation.

### 2.8 Data analysis

Data were processed using SPSS. Descriptive statistics summarized age, baseline classifications, participation, emotional perception and instrument scores. Quantitative variables were summarized using means and medians, while categorical variables were summarized using frequencies and percentages. Pre-post normality of score differences was assessed with the Shapiro-Wilk test. Because several paired differences were non-normally distributed and because a consistent approach across variables and zones was required, the Wilcoxon signed-rank test was used for paired comparisons. Statistical significance was set at α = 0.05. The primary pre-post analysis was complemented by geographic-zone analyses to examine consistency across central, northern and southern Tacna.

## 3 Results

### 3.1 Participant characteristics

In total, 217 older adults were enrolled in the study and all completed the 6-month intervention and post-intervention assessment. There was therefore 100% retention rate with no participant drop-out during the study period. Participants ranged in age from 61 to 95 years (*M* = 74.0 years, SD = 7.50).

### 3.2 Baseline cognitive and executive functioning by geographic zone

**Figure 1:**
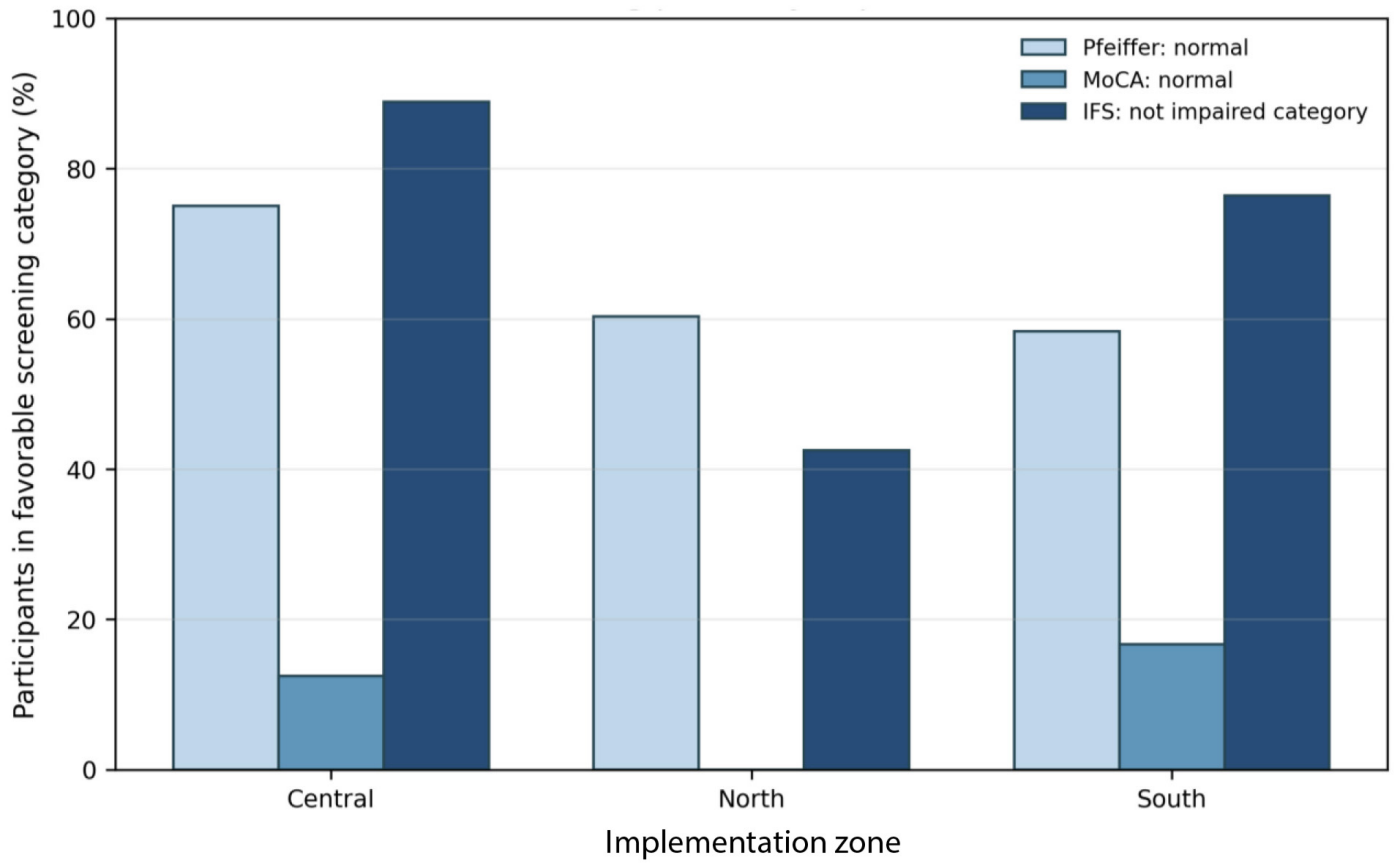
Baseline cognitive screening results stratified by implementation zone. This figure summarizes baseline territorial patterns to enhance clarity; detailed categorical frequencies are available in the source tables.

At baseline, the Pfeiffer Scale showed that normal cognitive status pre-dominated in all zones, although mild, moderate and severe impairment were also observed. MoCA screening revealed a higher prevalence of participants falling below the operational threshold, particularly within the northern zone. Concurrently, baseline IFS classifications highlighted executive-function vulnerabilities, which were most prominent in the northern region. These initial findings substantiate the necessity for a structured stimulation programme and offer a framework for interpreting subsequent outcomes. Baseline screening profiles by implementation zone are presented in Table 3. Baseline cognitive screening patterns by implementation zone are illustrated in Figure 1.

**Table 3:** Overview of baseline screening profiles stratified by implementation zone.

Zone	Pfeiffer (%)	MoCA (%)	IFS not-impaired category (%)	Interpretive
Central	75.0	12.5	88.9	Demonstrates relatively preserved cognitive status on the Pfeiffer scale; however, MoCA results indicate significant baseline cognitive vulnerability.
North	60.3	0.0	42.5	Characterized by the least favorable baseline profile across both MoCA and IFS screening assessments.
South	58.3	16.7	76.4	Presents a heterogeneous profile: low MoCA normality, yet IFS performance is less impaired relative to the northern zone.

IFS not-impaired category combines “maximum score” and “further assessment recommended” categories; results are screening classifications and not clinical diagnoses.

### 3.3 Pre-post changes in cognitive and executive scores

**Figure 2:**
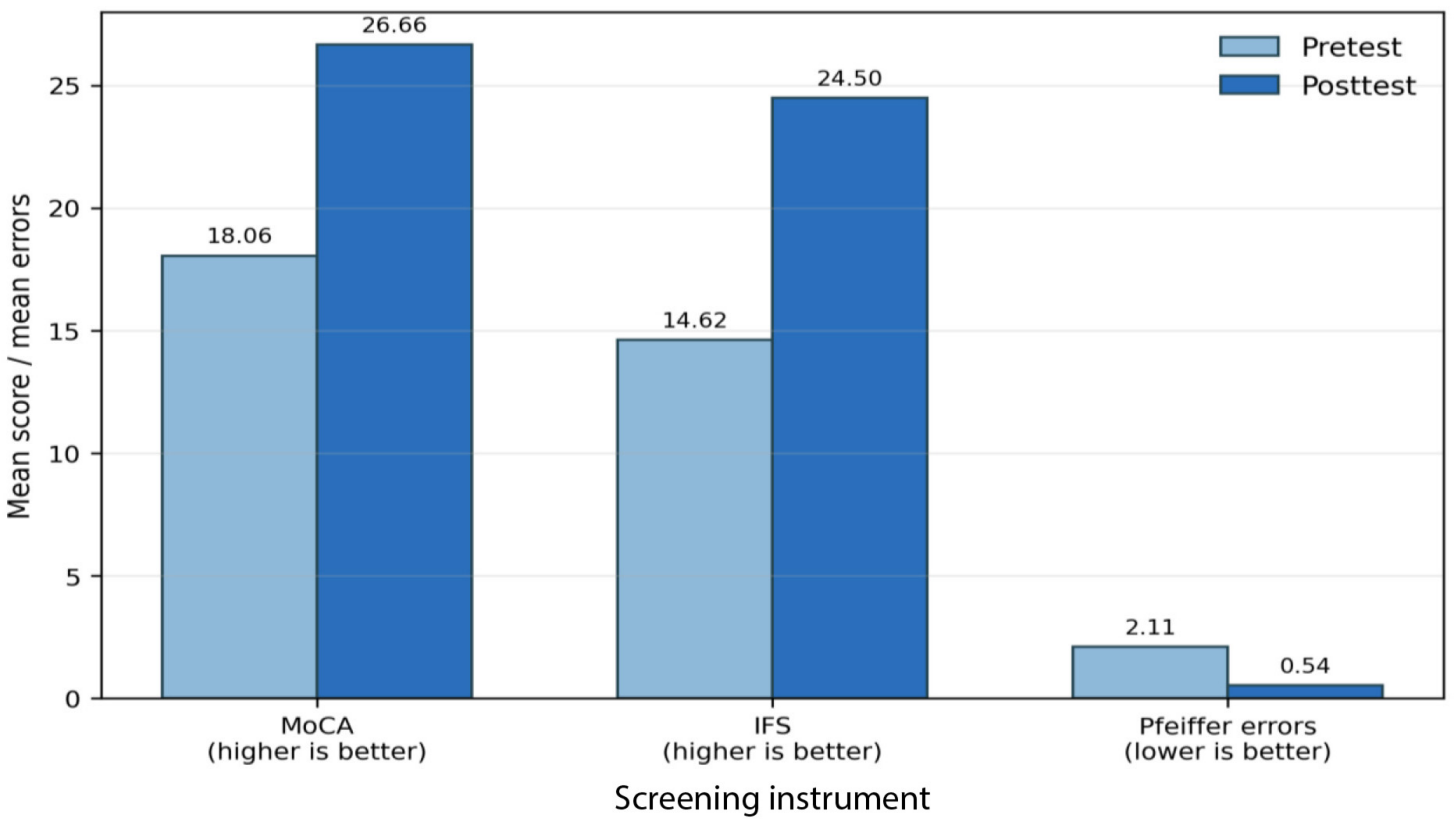
Pre-post change in cognitive and executive screening outcomes. The direction of improvement differs by instrument: higher MoCA and IFS scores indicate better performance, whereas lower Pfeiffer error counts indicate better cognitive status.

The intervention was associated with favorable change in all three cognitive-executive screening measures. Mean MoCA increased by 8.60 points, mean IFS increased by 9.88 points and mean Pfeiffer errors decreased by 1.57 errors. Median values showed the same direction of change, indicating that the observed pattern was not limited to mean scores. Detailed pre-post cognitive and executive screening changes are presented in Table 4. Pre-post changes in MoCA, IFS, and Pfeiffer outcomes are shown in Figure 2.

**Table 4:** Pre-post cognitive and executive screening changes with absolute differences.

Test	Pre mean	Post mean	Absolute change	Pre median	Post median	Wilcoxon V	*p*-value	Interpretation
MoCA	18.06	26.66	+8.60	18	27	6	< 0.001	Improved global cognitive screening performance.
IFS	14.62	24.50	+9.88	14	26	71.5	< 0.001	Improved executive-function screening performance.
Pfeiffer errors	2.11	0.54	−1.57	2	0	10,240	< 0.001	Reduced cognitive-error count.

Higher MoCA and IFS scores indicate better performance; fewer Pfeiffer errors indicate better cognitive status.

Wilcoxon tests compare paired pre-test and post-test scores.

The Wilcoxon signed-rank tests rejected the null hypothesis of no pre-post difference for MoCA, IFS and Pfeiffer scores (*p* < 0.001 in all cases). Therefore, the results support the study hypothesis that the programme would be associated with statistically significant improvement in cognitive status and executive functioning.

### 3.4 Consistency of pre-post changes by geographic zone

The secondary analysis by zone showed the same direction of change in central, northern and southern Tacna. MoCA and IFS scores increased after the intervention in all zones, while Pfeiffer errors decreased. All paired comparisons were statistically significant, suggesting territorial consistency of the pre-post improvement pattern. The zone-specific Wilcoxon signed-rank test results are shown in Table 5.

**Table 5:** Comparison of pre-post scores by geographic zone using the Wilcoxon signed-rank test.

Zone	Test	Wilcoxon V	*p*-value	Decision
Central	MoCA	2	2.475 × 10^−13^	*p* < 0.05; reject H_0_
Central	IFS	44.5	3.346 × 10^−12^	*p* < 0.05; reject H_0_
Central	Pfeiffer	544	1.965 × 10^−5^	*p* < 0.05; reject H_0_
North	MoCA	0	1.045 × 10^−13^	*p* < 0.05; reject H_0_
North	IFS	0	1.060 × 10^−13^	*p* < 0.05; reject H_0_
North	Pfeiffer	1,539.5	1.043 × 10^−9^	*p* < 0.05; reject H_0_
South	MoCA	1.5	2.571 × 10^−13^	*p* < 0.05; reject H_0_
South	IFS	1	2.517 × 10^−13^	*p* < 0.05; reject H_0_
South	Pfeiffer	1,530	1.820 × 10^−9^	*p* < 0.05; reject H_0_

The zone analysis was secondary and was used to evaluate consistency of the observed pattern across implementation settings.

### 3.5 Participation, acceptance and emotional perception

Before the intervention, participation in cognitive activities was classified as moderate in 60.4% of participants, low in 24.4% and high in 15.2%. After implementation, all participants were classified at a high level of post-intervention valuation of the workshops, indicating favorable acceptance of the cognitive stimulation activities.

**Figure 3:**
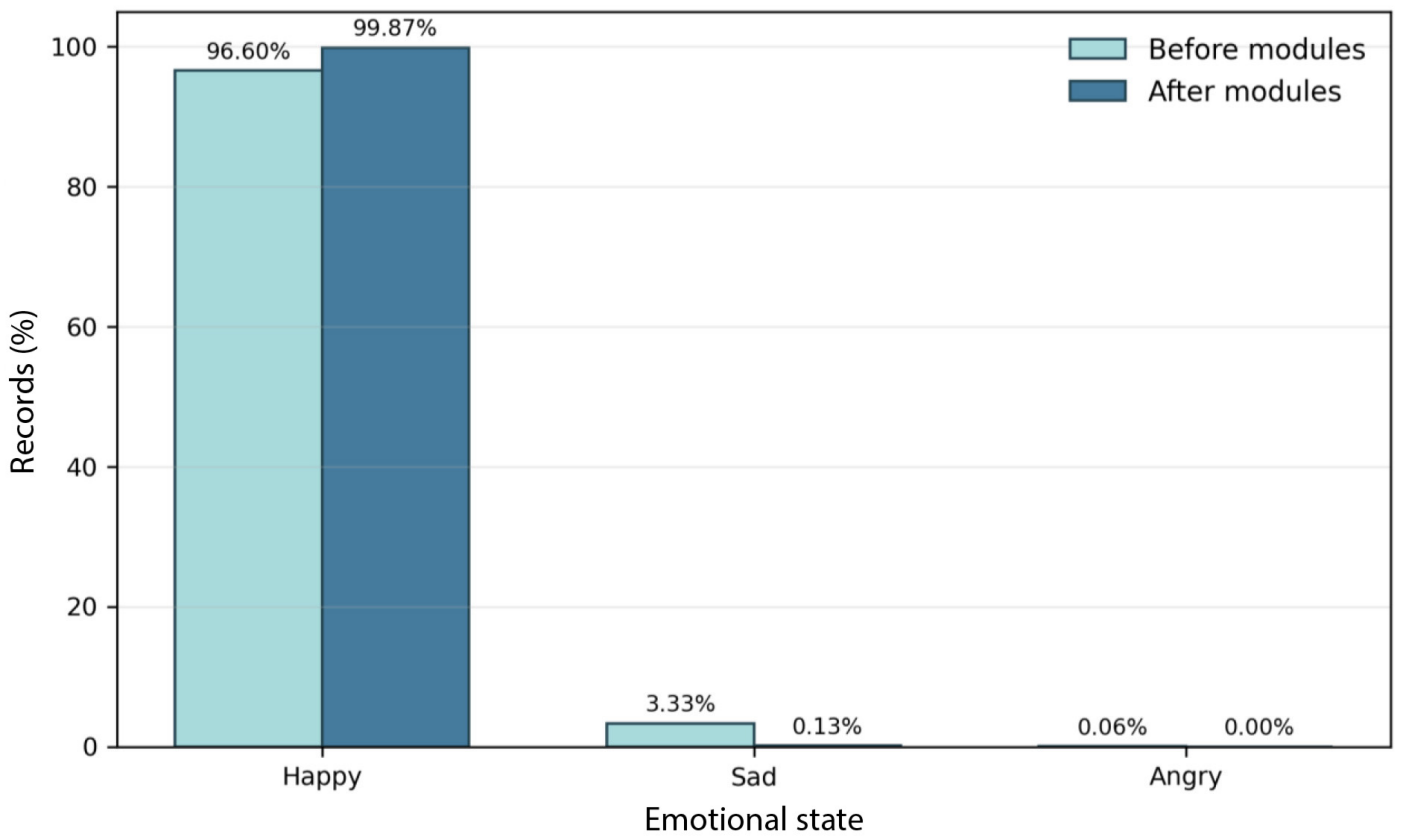
Immediate emotional perception before and after module activities. The high baseline percentage of “happy” responses indicates a ceiling effect; therefore, the emotional result is interpreted as favorable reception and stability, not as clinical evidence of affective improvement.

Emotional perception was measured using a categorical satisfaction form, administered immediately before and after each intervention session. Participants chose between three emotional states (happy, sad, or angry) providing the research team with the opportunity to monitor short-term emotional reactions associated with programme participation. Before activities, the “happy” category pre-dominated with 1,507 records (96.60%), followed by “sad” with 52 records (3.33%) and “angry” with one record (0.06%). After activities, “happy” increased to 1,558 records (99.87%), while “sad” decreased to two records (0.13%) and no “angry” responses were recorded. Across modules, 96.47% of emotional-perception records remained stable, 3.40% improved and 0.13% worsened. The distribution of immediate emotional perception before and after module activities is presented in Table 6. Immediate emotional perception before and after module activities is shown in Figure 3.

**Table 6:** General distribution of emotional perception before and after module activities.

Moment	Happy *n* (%)	Sad *n* (%)	Angry *n* (%)	Interpretation
Before modules	1,507 (96.60)	52 (3.33)	1 (0.06)	Very high positive baseline emotional perception.
After modules	1,558 (99.87)	2 (0.13)	0 (0.00)	Small favorable post-activity shift with ceiling effect.

Data correspond to module-level satisfaction records rather than independent diagnostic measures of emotional disorder.

All modules received favorable acceptance among participants who responded to the satisfaction item. The highest response frequencies were observed for graphomotor skills (97.70%), gnosias (92.63%), attention (89.86%) and language (89.40%). These results support the feasibility and acceptability of the intervention in participating community settings.

There was no participant attrition and high acceptance rates of all intervention modules, supporting the feasibility of implementing this cognitive stimulation programme in community settings serving older adults.

## 4 Discussion

This study found statistically significant pre-post improvement in three complementary screening outcomes after a 6-month cognitive stimulation programme for older adults in Tacna. MoCA and IFS scores increased, Pfeiffer errors decreased and the same direction of change was observed in central, northern and southern implementation zones. This coherent pattern supports the internal consistency of the findings and suggests that the programme was associated with short-term improvement in cognitive-executive screening performance.

The improvement in MoCA is clinically relevant as a screening signal because MoCA captures multiple cognitive domains, including attention, memory, language, abstraction, orientation and visuospatial-executive abilities. The improvement in IFS complements this finding by indicating change in executive processes such as inhibitory control, working memory, abstraction and motor programming. The reduction in Pfeiffer errors provides an additional brief indicator that participants made fewer orientation and memory-related errors after the programme. Taken together, these results justify the term “cognitive-executive functioning” and avoid the ambiguity of the previous term “mental functioning.”

The direction of the cognitive findings is consistent with the broader intervention literature. Yun and Ryu reported that cognitive-based interventions improved cognitive function in older adults in randomized controlled trials, with an overall effect size favoring intervention groups (9). The present study cannot claim the same causal strength because it lacked a control group, but its pre-post pattern is compatible with the expected direction reported in controlled evidence. These findings are also consistent with prior evidence from cognitive stimulation, cognitive training, and multidomain intervention trials showing benefits for cognitive outcomes and everyday functioning in older adults (11–14).

The intervention protocol offers a plausible mechanism for the observed changes because it repeatedly stimulated attention, memory, language, calculation, executive functions, gnosias, praxias and graphomotor skills through structured practice. The National Institute on Aging (3) suggests that cognitive training focused on enhancing specific skills may assist in maintaining cognitive health among older adults, a premise that validates the implementation of domain-targeted activities (3).

The National Institute on Aging (3) suggests that cognitive training focused on enhancing specific skills may assist in maintaining cognitive health among older adults, a premise that validates the implementation of domain-targeted activities.

Beyond individual exercises, the group-based structure likely fostered participant engagement and adherence. Drawing upon cognitive reserve theory, which posits that cognitively and socially stimulating pursuits may enable individuals to better withstand age-related or pathological brain changes (6), the program operated as more than a solitary exercise regimen. Instead, it established a sustained social-learning environment where older adults could practice tasks, exchange feedback, and engage with peers.

The importance of social participation is further supported by epidemiological evidence. Clare et al. (8) observed that a combination of cognitive and social activity, physical exercise, and healthy nutrition is positively correlated with cognitive function in later life, with cognitive reserve partially mediating this relationship. These findings suggest that community-based cognitive stimulation is most effective when it integrates structured cognitive demands with social interaction, rather than relying exclusively on isolated paper-and-pencil tasks.

The life-course perspective further strengthens the public-health relevance of the findings. Liu et al. (7) reported that cognitive reserve accumulated across early, middle and late life was associated with reduced dementia risk, including a protective effect in late life. Although the present intervention was short-term and cannot measure dementia incidence, it aligns with the idea that late-life cognitively and socially engaging activities may still be meaningful components of risk-reduction and healthy-aging strategies.

The emotional results should be interpreted with caution. The increase in “happy” responses from 96.60 to 99.87% and the reduction of sadness from 3.33 to 0.13% indicate favorable immediate emotional perception and programme reception. However, the very high baseline percentage of “happy” responses suggests a ceiling effect, and the satisfaction form is not equivalent to a validated depression, anxiety or psychological wellbeing scale. Therefore, the emotional finding should be described as acceptability and immediate positive perception rather than as clinical improvement in emotional health.

This cautious interpretation is important because cognitive impairment and dementia may be accompanied or preceded by changes in mood, emotional control, behavior or motivation (4). Future studies should therefore include validated affective and quality-of-life instruments if emotional wellbeing remains a primary outcome. Utilizing such instruments would enable the distinction between transient positive responses to activities, lasting mood changes, and clinically relevant psychological advantages.

Adding geographic-zone analyses adds further practical value for community-based interventions. Older adults living in different areas of Tacna might have different access to healthcare services, educational resources, transportation, social support networks and opportunities for community participation. This allowed the research team to examine how the cognitive stimulation programme performed across different social and community settings by looking at outcomes by zone. The large improvements across all three zones suggest the intervention may be transferable to different implementation settings and support its potential use in broader regional healthy-aging programmes.

These findings are pertinent to community health policy, The World Health Organization (5), emphasizes the importance of addressing modifiable risk factors due to the absence of curative treatments. A feasible programme that combines screening, structured cognitive stimulation, group participation and monitoring may therefore complement broader healthy-aging strategies in local settings, especially where access to specialized neuropsychological services is limited.

The main scientific contribution of the study is local and applied. It provides evidence that a structured cognitive stimulation programme can be implemented with older adults in Tacna and that it is associated with favorable pre-post changes in cognitive-executive screening measures. Its contribution is not to prove definitive efficacy, but to strengthen the empirical basis for improving, scaling and testing community cognitive-stimulation protocols under more rigorous designs.

### 4.1 Study limitations

Several limitations must be considered. First, the one-group pre-test-post-test design does not include an independent control group, so improvements cannot be attributed exclusively to the intervention because repeated testing, maturation, regression to the mean or contextual influences may have contributed to the observed changes. Second, participants were recruited from community settings and may not represent institutionalized, homebound or medically unstable older adults. Third, the dependence on the MoCA, Pfeiffer, and IFS as screening tools, rather than as comprehensive neuropsychological assessments, constrains the ability to conduct an in-depth analysis of domain specific recovery. Fourth, the evaluation of emotional wellbeing was performed using a concise satisfaction questionnaire centered on immediate perceptions, rather than through established clinical affective scales. Fifth, the acceptance of the module and participant engagement may be vulnerable to social desirability bias and the impact of the supportive relationships formed with the intervention team. Future research must emphasize the adoption of randomized controlled designs, include longitudinal follow up and employ validated psychological and comprehensive neuropsychological evaluations. Moreover, it is crucial to assess implementation metrics including intervention cost, facilitated fidelity, and adherence, these methodological enhancements would elucidate whether the observed improvements in screening performance are sustainable, clinically significant, and scalable within Peruvian community health systems.

## 5 Conclusion

The analysis indicates that the “Revitalizing Old Age” cognitive stimulation program produced statistically significant improvements in cognitive status and executive function before and after the intervention among older adults in Tacna, Peru. Significantly, mean MoCA scores increased from 18.06 to 26.66, while IFS scores enhanced from 14.62 to 24.50, simultaneously, the mean number of Pfeiffer errors decreased from 2.11 to 0.54. The Wilcoxon signed-ranks tests validated these improvements, confirming statistically significant differences between baseline and post intervention measures.

The applied program exhibited strong acceptability and a favorable trend in immediate emotional perception, with the percentage of participants indicating a “happy” state rising from 96.60 to 99.87 after module activities. Although these findings suggest feasibility and short-term enhancement, they lack definitive causal evidence due to the absence of a control group and a dependence on screening instruments. Future research should emphasize randomized controlled desgins, utilize validated affective assessments, and conduct longitudinal follow ups to ascertain the durability and clinical significance of these outcomes.

## Data Availability

The original contributions presented in the study are included in the article, further inquiries can be directed to the corresponding author.
